# Sex Differences in Onset and Progression of Cerebral Amyloid Angiopathy

**DOI:** 10.1161/STROKEAHA.122.040823

**Published:** 2023-01-24

**Authors:** Emma A. Koemans, Juan Pablo Castello, Ingeborg Rasing, Jessica R. Abramson, Sabine Voigt, Valentina Perosa, Thijs W. van Harten, Erik W. van Zwet, Gisela M. Terwindt, M. Edip Gurol, Jonathan Rosand, Steven M. Greenberg, Marianne A.A. van Walderveen, Alessandro Biffi, Anand Viswanathan, Marieke J.H. Wermer

**Affiliations:** 1 Department of Neurology, Leiden University Medical Center, the Netherlands (E.A.K., I.R., S.V., G.M.T., M.J.H.W.).; 2 Henry and Allison McCance Center for Brain Health, Massachusetts General Hospital, Harvard Medical School, Boston (J.P.C., J.R.A., J.R., A.B.).; 3 Department of Neurology, J Philip Kistler Stroke Research Center, Massachusetts General Hospital, Harvard Medical School, Boston (J.P.C., J.R.A., V.P., M.E.G., J.R., S.M.G., A.B., A.V.).; 4 Department of Neurology, University of Miami Miller School of Medicine, FL (J.P.C.).; 5 Department of Neurology, Otto-von-Guericke University, Magdeburg, Germany (V.P.).; 6 Department of Radiology, Leiden University Medical Center, the Netherlands (S.V., T.W.v.H., M.A.A.v.W.).; 7 Department of Biomedical Data Sciences, Leiden University Medical Center, the Netherlands (E.W.v.Z.).

## Abstract

Cerebral Amyloid Angiopathy (CAA) disease course is highly variable even in hereditary forms. Sex may be a possible modifying factor. We investigated biological sex differences in clinical disease course and magnetic resonance imaging-markers in sporadic (sCAA) and Dutch-type hereditary CAA (D-CAA).

**Methods:**

Patients with D-CAA and sCAA were included from hospital and research databases of the Leiden University Medical Center (2012–2020) and Massachusetts General Hospital (1994–2012). Key outcomes were: sex differences in symptomatic intracerebral hemorrhage (sICH) onset, recurrence and survival (analyzed using Kaplan Meier survival and regression analyses), and sex differences in magnetic resonance imaging-markers in D-CAA (explored using scatterplots), and in sCAA (investigated using regression analysis).

**Results:**

We included 136 patients with D-CAA (mean age 57 years, 56% women, 64% with previous sICH) and 370 patients with sCAA (mean age 76 years, 51% women, all with previous sICH). Men and women with D-CAA did not differ for sICH onset (median age 54 in men and 56 in women [*P*=0.13]). Men with D-CAA had a slightly higher number of sICH compared with women (median 2 versus 1; adjusted RR, 1.5 [95% CI, 1.1–1.9]) and a shorter interval between the first and second sICH (median 1.8 years for men and 3.1 years for women, *P*=0.02). Men with sCAA had their first sICH at an earlier age (median 75 versus 78 years, respectively, *P*=0.003) and more lobar microbleeds (median 1 versus 0, *P*=0.022) compared with women with sCAA. No substantial differences were found in the other magnetic resonance imaging markers. Survival after first sICH was comparable between sexes for D-CAA (*P*=0.12) and sCAA (*P*=0.23).

**Conclusions:**

Men with CAA seem to have an earlier onset (sCAA) and more hemorrhagic disease course (sCAA and D-CAA) compared with women. Future studies are necessary to confirm these findings and determine the underlying role of sex-related factors.

Cerebral amyloid angiopathy (CAA), caused by accumulation of the protein amyloid-β in the walls of the small cortical and leptomeningeal arteries, is thought to be present in 23% of the general population and in 48% of patients with Alzheimer disease.^1^ It is the cause of >50% of all lobar intracerebral hemorrhage (ICH) in the elderly, with the highest ICH recurrence rate and highest all-cause mortality of all primary ICH etiologies.^1–5^ Patients with CAA show a striking variability in clinical symptoms and disease course; some patients present with cognitive decline and have multiple lobar microbleeds on magnetic resonance imaging (MRI), while others suffer from transient neurological episodes or (repeated) lobar located symptomatic ICH (sICH).^2^ Even in Dutch-type hereditary CAA (D-CAA), an autosomal dominant variant of sporadic CAA (sCAA), there is a large variation in phenotype. Although patients carry the same causal mutation, the age at first sICH in patients with D-CAA ranges between 39 and 70 years and the number of sICH recurrences varies from 1 to 10.^6,7^

One possible contributing factor for this disease variability in CAA could be a modulating effect of sex. Until now, limited research has been performed investigating sex differences in CAA: a study in D-CAA from the early nineties reported higher mortality rates in women compared with men but these findings were never replicated.^8^ One study on primary ICH found that women have a higher risk of lobar ICH.^9^ However, in this study it was not clear whether the ICH was caused by CAA.

In contrast to CAA, the role of sex has been extensively examined in Alzheimer disease (AD). AD is also caused by amyloid-β accumulation and many AD patients show co-existent CAA pathology.^10^ Several studies have shown that ales with AD or mild cognitive impairment have more lobar microbleeds (a hallmark of CAA) compared with women.^11^ In contrast, AD occurs more frequently in women, and women with AD have higher levels of total amyloid-β load at histopathological examination.^12^ Some observational studies have suggested that estrogen may be protective against AD dementia and that its depletion could exacerbate AD progress in post-menopausal women.^12–14^ Based on these previous findings in D-CAA and AD, we hypothesize that sex might impact pathogenic pathways for amyloid-β deposition.

This study aims to explore the effect of biological sex in CAA on symptomatic ICH onset, sICH recurrence, survival, and the occurrence of CAA associated MRI markers in (pre)symptomatic D-CAA mutation carriers and patients with sCAA.

## Methods

We conducted an observational study in 2 cohorts: 1 with patients with D-CAA and 1 with patients with sCAA. Both cohorts contain clinical follow-up data and MRI data. Further details regarding the recruitment, inclusion, and follow-up of the participants can be found in the Supplemental Methods and Figure S1.

### Data Availability

Further information about the dataset can be obtained from the corresponding author upon reasonable request.

### Standard Protocol Approvals, Registrations, and Patient Consents

This study was approved by the local ethics review boards of the Leiden University Medical Center and the MGH, who waived the need for written informed consent from individual participants for the retrospective part of the study. The participants of the AURORA study and the patients from MGH who consented to longitudinal follow-up gave written informed consent for their participation in these studies.

### MRI

In the patients with D-CAA who participated in the prospective AURORA study, MRI scans of the brain were performed on a whole body human 3 Tesla MRI. The sCAA patients who were included in the MGH cohort were scanned with a 1.5 Tesla MRI.^4^ Further information regarding the scanners and MRI protocol can be found in the supplemental methods.

### Image Analysis

The following MRI markers were scored according to the STRIVE (Standards for Reporting Vascular Changes on Neuroimaging recommendations) criteria: lobar cerebral microbleeds, lobar macrobleeds, cortical superficial siderosis, enlarged perivascular spaces in the centrum semiovale (CSO-EPVS), and white matter hyperintensities (WMH).^15^ Criteria: lobar cerebral microbleeds were counted and scored on susceptibility weighted images or T2*-weighted images. Macrobleeds were also counted and scored on susceptibility weighted images or T2*-weighted images, T1 and T2-weighted images. Cortical superficial siderosis was scored on either susceptibility weighted images or T2*-weighted images using both the focality score and the hemisphere score, according to previously published classifications.^16,17^ CSO-EPVS were scored on T2-weighted images and classified into the following categories: no EPVS, 1–10, 11–20, 21–40, >40.^15,18^ WMH was graded separately for deep and periventricular white matter with the Fazekas score on fluid attenuated inversion recovery images.^15,19^ The total MRI brain burden of CAA-related small vessel disease score (CAA-CSVD) was calculated for each participant according to previously published methods.^20^

### Statistics

Statistical analyses were performed using the software R (R foundation for statistical computing, Vienna, Austria; www.R-project.org) and the Statistical Package for Social science (IBM SPSS). Figures were created using GraphPad Prism. Only participants with available data were used for the analysis, we did not perform data imputation. We used descriptive statistics to calculate means, medians and frequencies of the baseline characteristics of all participants. In patients with D-CAA, data from birth until date of medical file perusal (October 29, 2020) were included. This approach was chosen due to the genetic character of the disease and the uncertainty regarding the exact onset of the disease process. For this group we used age at October 29, 2020 or age at death if the participant was deceased before this time as the total follow-up time. All patients with sCAA first presented at the time of their first lobar sICH, and were followed from this moment onwards.

### Clinical Data

We used Kaplan Meier survival analysis with log-rank testing to investigate differences between men and women with CAA in age at first sICH and age at death. In the patients who suffered from at least 1 sICH we used Kaplan Meier survival analysis with log-rank testing to investigate differences between men and women in the survival time after the first sICH and in time between the first and the second sICH. Patients who died immediately after their first sICH (defined as death <3 weeks after 1st sICH) were included but censured in this analysis. All Kaplan Meier survival analysis were truncated when <10% of participants remained at risk. In D-CAA and sCAA patients who suffered from at least 1 sICH, we used Poisson regression analysis corrected for follow-up time after the first sICH to calculate adjusted rate ratios (aRR) with 95% CIs for the difference in total number of sICH between men and women. All analyses were performed for sCAA and D-CAA separately; no comparisons were done between the groups.

### MRI Data

We used descriptive statistics to calculate the proportion of symptomatic and presymptomatic men and women with D-CAA with CAA-related MRI markers (criteria: lobar cerebral microbleeds, lobar macrobleeds, cortical superficial siderosis, CSO-EPVS, WMH) and constructed explorative scatter plots to demonstrate the association of the markers with age at time of MRI scan. Because the number of D-CAA mutation carriers in the different age categories was limited, we refrained from performing formal statistical tests.

In patients with sCAA, we used binomial regression with an exponential link corrected for age at the time of MRI, APOE status, presence of hypertension and history of smoking to calculate adjusted odds ratios (aOR) with 95% CI for sex differences in presence of lobar criteria: lobar cerebral microbleeds, presence of cortical superficial siderosis, presence of >20 CSO-EPVS, presence of >40 CSO-EPVS, and presence of deep and periventricular WMH on MRI performed <3 months after sICH. In patients with sCAA we used Poisson regression analysis corrected for age at time of MRI, APOE status, presence of hypertension and history of smoking to calculate aRR with 95% CI for sex differences in number of lobar criteria: lobar cerebral microbleeds and CAA-CSVD score, scored on MRI performed <3 months after sICH.

## Results

### D-CAA

**Figure 1.**
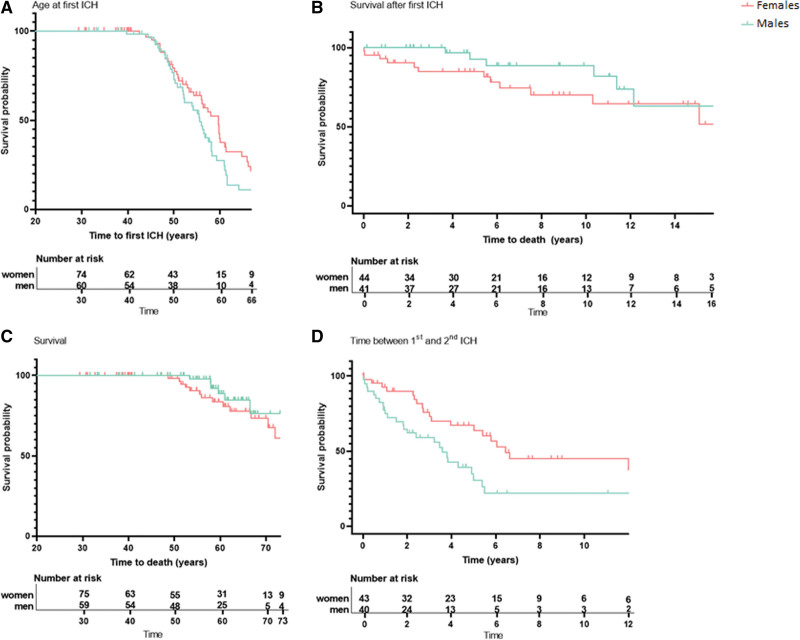
**Symptomatic intracerebral hemorrhage (sICH) onset, time between first and second sICH, and survival in men and women with Dutch-type hereditary cerebral amyloid angiopathy (D-CAA). A**, Difference in age at first sICH between men and women with D-CAA. N=60 men, 75 women (1 patient excluded from analysis as age at first sICH was unknown). *P*=0.13 (log rank). **B**, Difference in survival after first sICH between men and women with D-CAA and at least 1 sICH. N=41 men, 43 women (2 patients excluded as death was not cerebral amyloid angiopathy [CAA] related). *P*=0.12 (log rank). Including the patients whose death was not CAA-related did not significantly change the outcome (*P*=0.14). **C**, Difference in age at death between men and women with D-CAA. N=59 men, 75 women (2 patients excluded as death was not CAA-related). *P*=0.27 (log rank). Including the patients whose death was not CAA-related did not significantly change the outcome (*P*=0.34). **D**, Difference in time between first and second sICH between men and women with D-CAA. N=40 men, 43 women (3 patients excluded because age at second sICH was unknown, patients who died within 3 wk of first sICH (n=2) included in analysis but censured). *P*=0.02 (log rank).

We included 136 patients with D-CAA (mean age 57 years, 56% women). Eighty-seven (64%) of the 136 patients had a history of sICH (70% of the men and 59% of the women). Men with D-CAA had their first sICH at a median age of 54 years (range 40–72), and women with D-CAA at a median age of 56 years (range 42–78; *P*=0.13; Figure 1A). During follow-up (mean follow-up time 58 years [range 29–87]), 7 (12%) of the men and 14 (18%) of the women with D-CAA died. All deaths except 2 were caused by CAA-related ICH; these 2 participants were excluded from the survival analysis. Survival after the first sICH was comparable between men and women with D-CAA: median of 8 years (range 4–12) for men and 5.4 years (range 0–19) for women (*P*=0.12; Figure 1B), as was overall survival: median survival of 59 years (range 53–67) for men and 56 years (range 49–72) for women (*P*=0.27; Figure 1C). Men with D-CAA had a higher number of recurrent sICH compared with women (median number of sICH 2 [range 1–9] for men and 1 [range 1–5] for women, aRR 1.5, [95% CI, 1.1–1.9]; *P*=0.01). Median time between the first and second sICH was 1.8 years for men with D-CAA and 3.1 years for women (*P*=0.02; Figure 1D).

**Figure 2.**
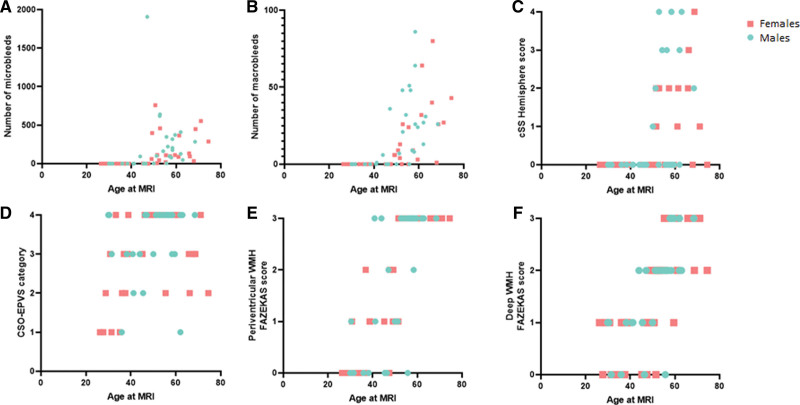
**Magnetic resonance imaging (MRI) markers in men and women with Dutch-type hereditary cerebral amyloid angiopathy.** WMH indicates white matter hyperintensities.

Sixty-four (mean age 50 years, 56% women) of the 136 patients with D-CAA participated in the prospective AURORA study. Frequencies of the different MRI markers are shown in Table 1 and Figure 2. Occurrence of CSO-EPVS and WMH on MRI were the earliest CAA-related MRI markers in both pre-symptomatic men and women with D-CAA (Table 1, Figure 2, Figure S2). The proportion of the different MRI markers in our exploratory analyses seemed to be comparable between men and women with D-CAA over age (Figure 2), although some men had a relatively high number of macrobleeds and cortical superficial siderosis at a relative early age (Figure 2B and 2C).

**Table 1.**
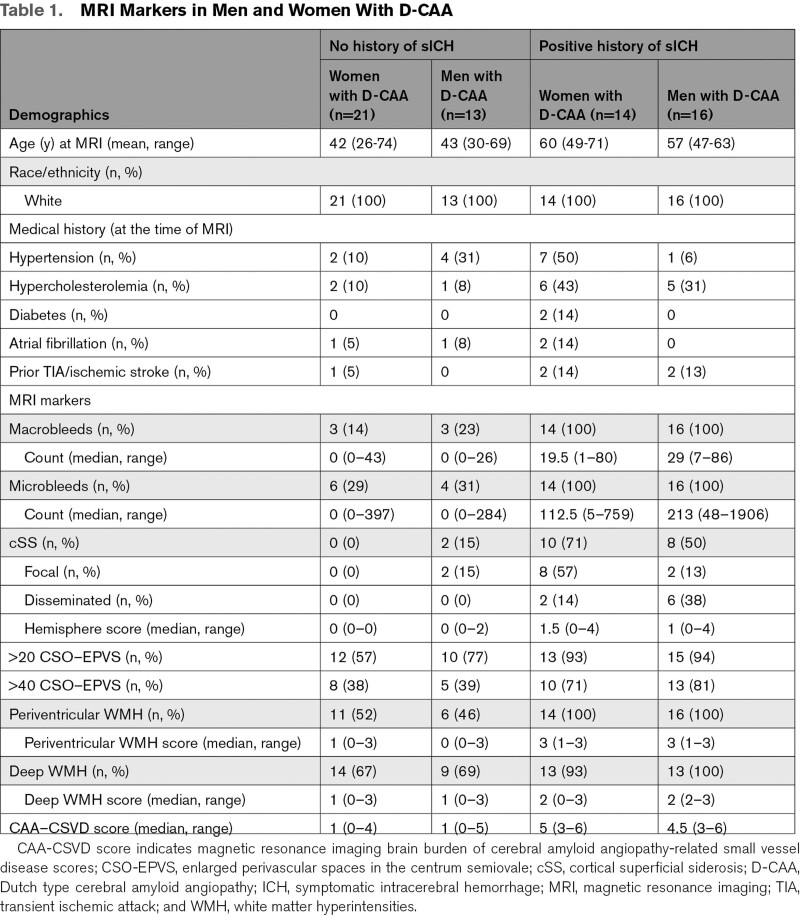
MRI Markers in Men and Women With D-CAA

### sCAA

We included 370 participants with sCAA (mean age at sICH 76 years, 51% women): 151 were diagnosed with probable CAA (89 women and 62 men) and 219 with possible CAA (106 women and 113 men) based on the modified Boston criteria. All had a history of sICH and were followed from the moment of their first ICH onwards: average follow-up time was 71.2 months (interquartile range 44.7–88.6).

**Figure 3.**
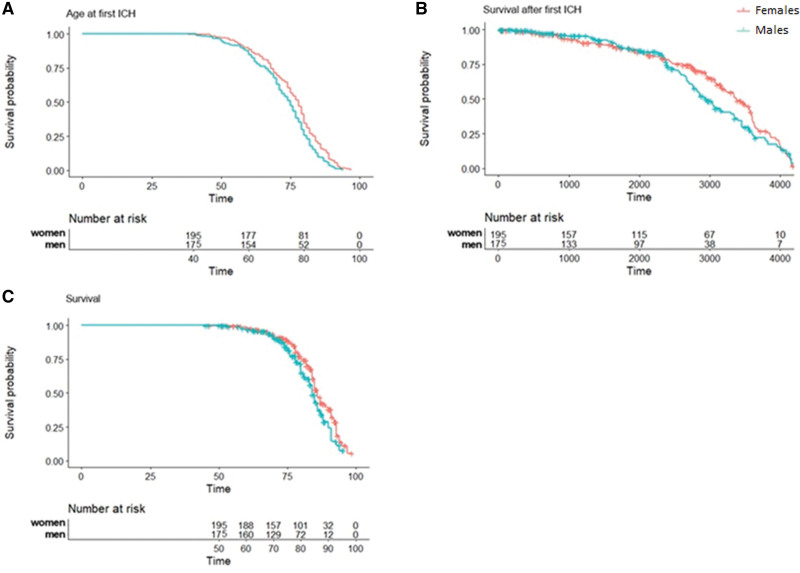
**Symptomatic intracerebral hemorrhage (sICH) onset and survival in men and women with sporadic cerebral amyloid angiopathy (sCAA). A**, Difference in age at first sICH between men and women with sCAA. Time in years. *P*=0.003 (log rank). **B**, Difference in survival after first sICH between men and women with sCAA. Time in days. *P*=0.23 (log rank). **C**, Difference in overall survival between men and women with sCAA. Time in years. *P*=0.03 (log rank).

Men with sCAA had their first sICH at a median age of 75 years (range 41–95) and women with sCAA at a median age of 78 years (range 38–97), log-rank test *P*=0.003 (Figure 3A). During follow-up, 79 (45%) of the men and 94 (48%) of the women with sCAA died. Men with sCAA died at a median age of 80 years (range 53–94) and women with sCAA at a median age of 83 years (range 47–97), log-rank test *P*=0.03 (Figure 3C). Overall survival after the first sICH between men and women, however was comparable (*P*=0.23; Figure 3B). In total, there were 91 sICH recurrent events during follow-up, 48 in men and 43 in women with sCAA. There was no difference in ICH recurrence between men and women (*P*=0.23). Frequencies of the different MRI markers are shown in Table 3. Men had significantly more often lobar microbleeds compared with women (*P*=0.006), and a higher median lobar microbleed count (median count of 1 in men versus 0 in women, *P*=0.02). The frequencies of the other MRI markers as well as the CAA-CSVD scores were comparable between men and women with sCAA.

## Discussion

We found that men with D-CAA had a higher number of recurrent sICH and a shorter time between the first and second sICH compared with women. In patients with sCAA, men had their first sICH at an earlier age than women, and men had more microbleeds on MRI compared with women. Overall, men with CAA seemed to have an earlier disease onset (sCAA) and a more hemorrhagic disease course (D-CAA and sCAA) compared with women. Survival after first sICH was comparable between sexes for both D-CAA and sCAA.

The biological sex differences in CAA increase our understanding of CAA pathophysiology and have implications for clinic and research. With our results, patients can be better informed about the influence of sex differences on the disease course. Also, the suggestion of a more hemorrhagic phenotype in men with CAA might be a factor to consider in decisions regarding (re)start of anticoagulation. The mechanisms behind the sex-differences could be possible future targets for prevention and treatment of CAA. A possible driving force behind the differences could be the influence of (women) sex-specific factors such as (epi)genetic factors and sex-hormones. Previous studies in Alzheimer disease have shown that estrogen (depletion) possibly influences disease course, including the accumulation of the amyloid-β peptide in brain tissue, and that the hormone might protect against Alzheimer disease.^13,14,21,22^ Due to the sex-differences found in this current study, which seem protective for women, one could hypothesize that the protective effect of estrogen also plays a role in vascular amyloid accumulation, although this has not yet been investigated. This could especially be the case in D-CAA, where women can be affected at a relatively young pre-menopausal age. However, also in sCAA it can be hypothesized that estrogen plays a protective role. Previous research has shown that inflammation is abundantly present in brain tissue of patients with CAA, and is related to microbleed development in CAA-mouse models.^23–25^ Estrogen has anti-inflammatory effects, which might be a second possible pathway for the influence of estrogen on CAA disease course and CAA related ICH.^26–28^ Another explanation for the sex differences in our study could be residual confounding by differences in comorbidity or (exposure to) unidentified (epi) genetic or environmental vascular risk factors. Future studies are necessary to determine whether there is a link between female sex (hormones) and CAA, for instance by investigating the effect of pregnancy and menopause (onset) and use of hormone suppletion on CAA and to investigate sex differences in histopathology, CAA animal models or organ-on-chip models.^29^

A previous study from the nineties found higher overall mortality in women with D-CAA compared with men.^8^ This finding could not be reproduced in our study although median survival in women with D-CAA was shorter than in men. However, because this difference was not statistically significant it is most likely that this finding is due to chance. Alternatively, the lack of significance could be caused by the relatively small sample size and point toward a true difference in survival. It is not likely that the difference in survival was the cause of the found difference in sICH recurrence, as we adjusted for follow-up time in the analysis. Hypothetical explanations for a possible difference in survival are a more detrimental ICH course in women or a higher risk of misdiagnosis with consequent delays of acute stroke treatment, as has been recently shown to be the case in ischemic stroke.^30^ Our current study included less participants than the previous study and the overall mortality was lower. It is, therefore, possible that we did not have enough power to detect differences in mortality. It is also possible that due to improvement of overall clinical care and post-ICH rehabilitation compared with the nineties previously found differences in survival might have decreased. Lastly, our D-CAA database only includes patients who presented at the (outpatient) clinic, and is therefore prone to “immortal time bias.” Patients need to be alive to be able to present themselves at the hospital and those who die due to their first sICH at home can therefore not be included. Future studies are necessary to further investigate sex-differences in survival and ICH recurrence, and the possible underlying mechanisms.

CSO-EPVS and WMH were the earliest MRI markers in both men and women with D-CAA. Macrobleeds and microbleeds only manifest themselves after the age of 40, comparable to what was found in previous studies regarding MRI markers in presymptomatic and symptomatic D-CAA.^31,32^ We found a remarkable variability in the proportions of CAA-related MRI markers within patients of the same age category (Figure 2). This suggest that other genetic and/or life-style factors might influence CAA development in D-CAA. APOE status was not available for our D-CAA participants, however an earlier study did not identify an effect of APOE status on MRI markers (white matter hyperintensities) in D-CAA.^33^

Our study has limitations. Firstly, the follow-up data of D-CAA patients were in part retrospectively collected and therefore some events could have been missed. This may have also caused a selection bias, as patients who did not report at the Leiden University Medical Center could not be included in the study. The radiological data of both the sCAA and D-CAA populations were derived from a prospective study, in which all participants underwent the same MRI protocol. However, the number of participants with D-CAA in the different age groups was small and included relatively healthy patients, again possibly causing selection bias. Therefore we refrained from performing statistical analyses on the MRI markers in this group, and consider that part of our study explorative. Another limitation of this study is the inclusion of both possible and probable sCAA patients. Both groups were included to cover early and advanced stages of CAA and to be able to generate enough power to enable statistical comparisons. However, the addition of patients with possible CAA could have diluted our findings because of possible misclassification. Lastly, we chose in this first explorative study to only include sCAA patients with a history of ICH. sCAA has a broad spectrum of symptoms, and there are patients with sCAA who never suffer from an ICH. We chose to investigate those with ICH to enable better comparison to the D-CAA population, who are known to all eventually suffer from ICH. Future research is needed to investigate sex-differences in nonhemorrhagic symptoms such as dementia and cognitive impairment in CAA.

The strength of our study lies in the investigation of sex differences in both a unique hereditary and a sporadic cohort of CAA patients. In contrast to sCAA, for which only a possible or probable diagnosis can be made during life, D-CAA can be diagnosed with absolute certainty by genetic testing.^34^ Furthermore, as D-CAA occurs at a relatively young age, patients have far less age-related small vessel disease. Therefore, D-CAA is considered to be a relatively “pure” form of CAA in which the early and presymptomatic stages of CAA can be investigated. We chose in this study to not combine or compare the results between sCAA and D-CAA cohorts directly, as the baseline characteristics of these cohorts are too different for a direct comparison: the cohorts differ not only in age and age-related cardiovascular risk factors, but also in race/ethnicity (Tables 1 and 2), and MRI field strength and protocol. That, despite these differences, results of this study point into the same direction for both D-CAA and sCAA strengthens our findings.

**Table 2.**
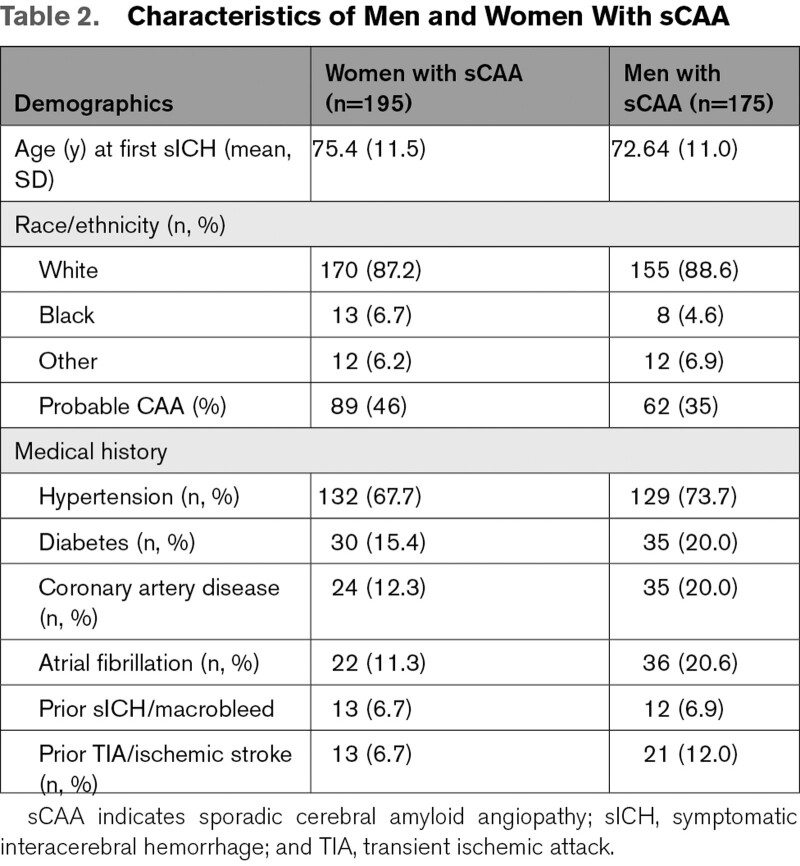
Characteristics of Men and Women With sCAA

**Table 3.**
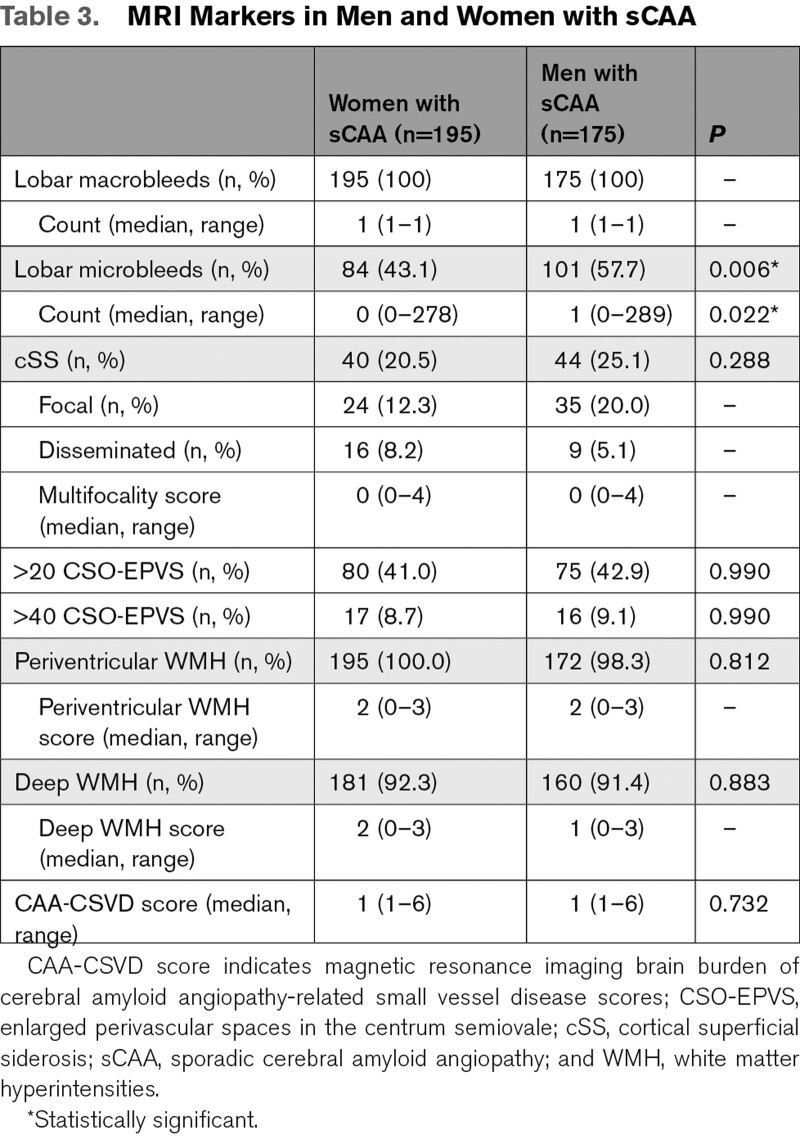
MRI Markers in Men and Women with sCAA

To conclude, our study suggests that sex influences on the disease course of patients with D-CAA and sCAA may be present. Our results seem to show that male sex is possibly associated with an earlier onset in sCAA, and a more hemorrhagic disease course in sCAA and D-CAA. Future prospective studies are necessary to confirm these findings and determine which sex related factors could be important for CAA progression, and could therefore be a possible target for CAA prevention and treatment.

## Article Information

### Sources of Funding

This work was supported by an Clinical Established Investigator grant of the Netherlands Heart Foundation 2016T086 to Dr Wermer, by the Dutch CAA foundation and by the American Heart Association-Bugher Foundation, and by NIH K23NS100816. The funding agency had no role in the design or conduct of the study.

### Disclosures

Dr Koemans reports independent support in the form of travel grants from Alzheimer Nederland, the Dutch Heart foundation and Academy Van Leersum grant of the Academy Medical Sciences Fund, Royal Netherlands Academy of Arts & Sciences, and from the Dutch CAA foundation. Dr Terwindt reports independent support from de Nederlandse Organisatie voor Wetenschappelijk Onderzoek, European Community, the Dutch Heart Foundation, the Dutch Brain Foundation, and the Dutch CAA foundation. Dr Gurol reports grants received from AVID, Pfizer, and Boston Scientific. Dr Rosand reports independent support from the US National Institutes of Health, the American Heart Association, and consulting for Takeda Pharmaceuticals and the National Football League. Dr Greenberg reports independent support from the US National Institutes of Health. Dr Viswanathan reports consulting for Biogen and Roche. Dr Wermer reports independent support from de Nederlandse Organisatie voor Wetenschappelijk Onderzoek ZonMw (VIDI grant 91717337), the Netherlands Heart Foundation, and the Dutch CAA foundation. The other authors report no conflicts.

### Acknowledgments

Conducted by Drs Koemans, van Zwet, Castello, Perosa, and Biffi.

### Supplemental Material

Supplemental Methods

Figures S1–S2

STROBE checklist

## Supplementary Material
