# Supplementary material for: Sex Differences in Onset and Progression of Cerebral Amyloid Angiopathy
Source: Stroke. 2023 Jan 24;54(2):306–14. doi: 10.1161/STROKEAHA.122.040823 (PMC9855754; doi:10.1161/STROKEAHA.122.040823)

SUPPLEMENTAL MATERIAL

Supplemental methods

Participants

In this study we investigate the effect of biological sex on CAA. We do not investigate the effect of gender. All participants are cis-gender individuals who did not go through medical or social transitioning.

The participants with D-CAA were derived from a retrospective D-CAA database that included all consecutive D-CAA mutation carriers who visited the Leiden University Medical Center (LUMC) neurology (outpatient) clinic between January 2012 and November 2020. D-CAA was diagnosed 1) by genetic testing for the causal mutation on codon 693 in the APP gene or 2) when patients had a history of ≥ 1 lobar sICH with features of CAA according to the modified Boston criteria on MRI and ≥ 1 first-degree relative diagnosed with D-CAA. Both pre-symptomatic and symptomatic (defined as a history of sICH) D-CAA mutation carriers were included. Symptomatic ICH was defined as acute onset of symptoms which could be attributed to an ICH visible on radiological imaging. The database contains information on demographics, clinical characteristics and disease outcome (age at first sICH, sICH recurrence and survival). We obtained date and cause of death by reviewing clinical charts and, if necessary, consulting the general practitioner. A subset of the mutation carriers included in the D-CAA database also participate in the ongoing prospective D-CAA natural history study (called the AURORA study). Participants for this study were recruited via the (outpatient) clinic of the LUMC. This follow-up study started in 2018 and aims to investigate CAA disease course and to identify new CAA related biomarkers in (pre)symptomatic D-CAA. All AURORA participants underwent yearly MRI and were interviewed in-person on demographics, medical history and clinical symptoms. MRIs were

performed between 2018-2020 and scored by a single observer with>5 years of experience in the field (EAK).

The participants with sCAA were derived from the Massachusetts General Hospital (MGH) sCAA database that contains prospectively collected data from a longitudinal cohort of sCAA patients who visited the MGH in Boston between January 1994 and December 2012. For this study we only included patients with possible or probable sCAA according to the modified Boston criteria who had a history of lobar sICH; we excluded participants with inflammatory CAA. Information on demographics, medical history and clinical symptoms was obtained through in-person interviews. Follow-up data were collected for those patients who consented to a longitudinal follow-up via telephone calls at 3 months after enrollment and every 6 months thereafter. Patients were followed until the moment of data collection for this paper, which was August 2021. Patients underwent MRI scans performed within 3 months after lobar sICH. MRIs were performed between 1994-2012, scored by multiple experienced observers and checked by a single observer with >10 years of experience in the field (AV). See supplemental figure 1 for a flow-chart detailing inclusion of the participants with D-CAA and sCAA.

This manuscript is reported against the STROBE guidelines.

MRI

In the patients with D-CAA who participated in the prospective AURORA study, MRI scans of the brain were performed on a whole body human 3 Tesla (3T) Philips Achieva MRI scanner (Philips Healthcare, Best, The Netherlands). The data were acquired using a standard 32-channel head coil. The following sequences were performed: Three-dimensional T1

weighted images, T2 weighted images, three dimensional Fluid Attenuated Inversion Recovery (FLAIR) images, and susceptibility weighted images (SWI). The sCAA patients who were included in the MGH cohort were scanned with a 1.5T GE Sigma MRI scanner (GE Healthcare, Chicago, IL, USA). The following sequences were performed: a whole brain axial T2-weighted, T2*-weighted gradient-recalled echo or SWI, FLAIR images and T1-weighted sequences.

Supplemental figure 1: Flowchart

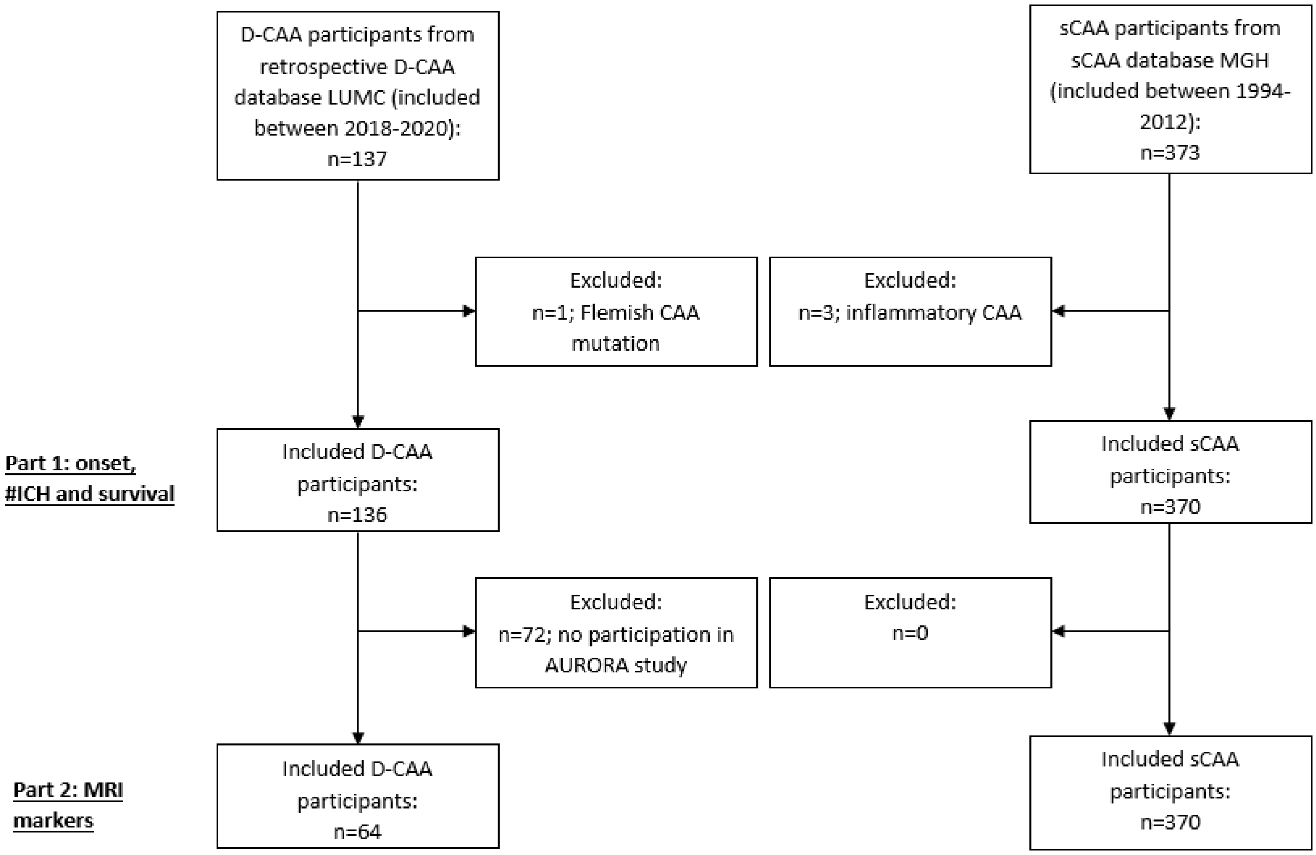

Supplemental figure 2: CAA-CSVD burden on MRI in D-CAA

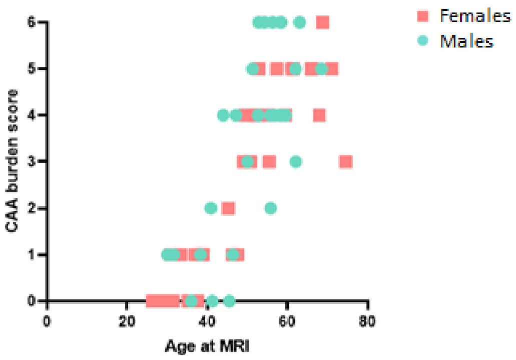

Supplement: Supplementary file 1 [file str-54-306-s001.pdf]
